# Implications of Rectal Cancer Radiotherapy on the Immune Microenvironment: Allies and Foes to Therapy Resistance and Patients’ Outcome

**DOI:** 10.3390/cancers15215124

**Published:** 2023-10-24

**Authors:** Dorothée Sartorius, Moritz Leander Blume, Johannes Robert Fleischer, Michael Ghadimi, Lena-Christin Conradi, Tiago De Oliveira

**Affiliations:** Department of General, Visceral and Pediatric Surgery, University Medical Center Göttingen, Robert-Koch-Straβe 40, 37075 Göttingen, Germany; dorothee.sartorius@stud.uni-goettingen.de (D.S.); moritz.blume@stud.uni-goettingen.de (M.L.B.); j.fleischer01@stud.uni-goettingen.de (J.R.F.); mghadimi@med.uni-goettingen.de (M.G.)

**Keywords:** rectal cancer, radiotherapy, chemoradiotherapy, tumor immune microenvironment, radioresistance

## Abstract

**Simple Summary:**

The efficiency of (chemo-)radiotherapy for rectal cancer is not only determined by the impact on the tumor cells themselves but also by the highly individual surrounding tumor microenvironment, including immune cells. However, many aspects of the radiation-induced immune response remain to be fully understood. This review summarizes existing literature about the effects of (chemo-)radiotherapy on the rectal cancer immune microenvironment, which can be both tumor-suppressive or pro-tumorigenic, by either promoting an effective anti-tumor immune response or mediating resistance. We further aim to highlight potential immune-modulating combination therapies, such as immune checkpoint inhibitors, that offer individualized approaches to target the heterogeneous tumor immune microenvironment.

**Abstract:**

Aside from surgical resection, locally advanced rectal cancer is regularly treated with neoadjuvant chemoradiotherapy. Since the concept of cancer treatment has shifted from only focusing on tumor cells as drivers of disease progression towards a broader understanding including the dynamic tumor microenvironment (TME), the impact of radiotherapy on the TME and specifically the tumor immune microenvironment (TIME) is increasingly recognized. Both promoting as well as suppressing effects on anti-tumor immunity have been reported in response to rectal cancer (chemo-)radiotherapy and various targets for combination therapies are under investigation. A literature review was conducted searching the PubMed database for evidence regarding the pleiotropic effects of (chemo-)radiotherapy on the rectal cancer TIME, including alterations in cytokine levels, immune cell populations and activity as well as changes in immune checkpoint proteins. Radiotherapy can induce immune-stimulating and -suppressive alterations, potentially mediating radioresistance. The response is influenced by treatment modalities, including the dosage administered and the highly individual intrinsic pre-treatment immune status. Directly addressing the main immune cells of the TME, this review aims to highlight therapeutical implications since efficient rectal cancer treatment relies on personalized strategies combining conventional therapies with immune-modulating approaches, such as immune checkpoint inhibitors.

## 1. Introduction

Colorectal cancer (CRC) represents the third most diagnosed cancer and the second leading cause of cancer mortality for both sexes combined, comprising approximately 1 in 10 cancer cases and deaths worldwide in 2020 [1]. While the incidence of CRC for ≥50 year-old adults showed a favorable trend in recent years, the incidence recently increased in the group of young adults (<50 years-old) in multiple high-income countries [1,2,3,4].

The standard treatment for locally advanced rectal cancer (LARC) involves a multimodal approach commonly including neoadjuvant chemoradiotherapy (nCRT) or short-course radiotherapy (SCRT), total mesorectal excision and adjuvant chemotherapy (CT) [5]. SCRT generally involves five fractions at a dosage of 5 Gy within one week, followed by early surgery, while conventional nCRT is usually administered in a long-course (LCRT) regimen of 1.8–2 Gy fractions, leading up to a total dosage of 45–50.4 Gy before delayed surgery [6,7,8]. The recommended chemotherapy agents used within nCRT regimens for rectal cancer are typically capecitabine or 5-fluorouracil (5-FU) [5,9,10]. Although improvement of local tumor control was described as the main advantage of neoadjuvant radiotherapy (nRT) and nCRT, an increase in overall survival (OS) could not be demonstrated by their administration [8,11,12]. Recently, total neoadjuvant therapy (TNT) has evolved into a promising novel approach for rectal cancer therapy, combining CRT and systemic CT prior to surgery [13,14,15]. As potential benefits of TNT, in comparison to standard CRT for LARC, an increase in pathological complete response (pCR), disease-free survival and OS as well as reduced risk for distant metastasis have been reported [16]. Radiation is a crucial part of combination therapy strategies for rectal cancer and it is imperative to continuously improve its efficiency. Yet, the response to (C)RT still varies widely amongst patients treated for rectal cancer, and the molecular mechanisms underlying this individuality as well as potential predictors remain to be fully understood [17,18]. Consequently, additional research is required to investigate the effects of radiation on rectal cancer and its contribution to mechanisms of treatment resistance.

Over the past two decades, the perception of cancer has shifted from a view centering the cancer cell to a concept embedding it in a network of stromal components such as fibroblasts, endothelial cells and a variety of immune cells, summarized as the tumor microenvironment (TME) [19,20]. The diverse TME has been recognized as an important factor in tumor progression and metastasis and represents an attractive target for therapy approaches [21,22]. Additionally, increasing attention has been directed towards elucidating its role in resistance against systemic therapies [23] and RT [18,24].

Colon and rectal cancer, even though being highly related, can be considered distinct diseases [25,26]. Importantly, their difference also reflects in the tumor immune microenvironment (TIME) [27,28]. An important molecular difference between colon and rectal cancer is the gradient in microsatellite instability, which was detected by Salem et al. in 22.3% of right-sided colon tumors, 7.1% of left-sided colon tumors, but only in 0.7% of rectal cancers [29]. In a study by Lin et al., microsatellite instability-high CRC showed a more favorable response to immunotherapy and enhanced immunogenicity in comparison to microsatellite stable CRC, which was observed to be prone to an inhibitory TME [28]. Additionally, the authors speculated that microsatellite instability-high colon and rectal cancer might exhibit differences in their TME and immunologic features [28]. Consistent with these findings, Mezheyeuski et al. concluded from the comparison of immune cell compositions in pre-treated tumor samples that the level of natural immune activation seems to be lower in rectal than in colon cancer [27]. Therefore, it is crucial to consider the disparities between the TIME of rectal and colon cancer when conducting cancer therapy research.

In line with the former cancer-cell-centered understanding of malignant tumors, for decades, the impact of RT on the cancer cells’ DNA was the main focus of research, neglecting its interconnected effects on the TME, such as inflammation, immunomodulation, and extracellular matrix remodeling [24,30]. However, several previous studies suggested that RT and CRT might have stimulating effects on the rectal cancer TIME, as seen in altered infiltration of lymphocytes and dendritic cells (DCs) [31,32,33,34,35,36]. Yet, (C)RT might also prompt immunosuppressive changes in the rectal cancer TIME by decreasing the number of B cells [31] or upregulating the programmed cell death ligand 1 (PD-L1) [37,38]. Irradiated rectal cancer cells were also shown to induce immune escape mechanisms to circumvent phagocytosis and immune recognition [39]. Thus, immunological effects of radiation within the TME can lead to resistance and recurrence in multiple manners, making the combination of RT and immunotherapy a promising therapeutical approach [24].

This review focuses on the effects of radiation on the rectal cancer TIME, whilst emphasizing the distinct roles of immune cells. Nevertheless, considering the current clinical practice, where RT is often combined with CT, we have expanded our literature review to include the effects of CRT. Further, even though their differences are increasingly recognized, colon and rectal cancer are still commonly combined as single entity based on anatomical, functional, and histological similarities [26]. Therefore, since rectal cancer-specific literature on certain topics remains limited, we considered it necessary to occasionally reference results from CRC studies in order to complement findings in the context of rectal cancer or propose potential avenues for further rectal cancer-specific investigation.

## 2. (Chemo-)Radiotherapy’s Dual Impact on the Rectal Cancer TIME: Immuno-Activating and -Suppressive Effects and Their Therapeutic Implications

Multiple studies have indicated that nCRT can increase the level of immune activation within the rectal cancer TIME via alteration of immune cell infiltration and effector activity, potentially associated with enhanced anti-cancer responses [31,32,33,34,35,36]. However, radiation can also trigger processes within the TIME that can lead to cancer progression or relapse, many of which are associated with immunosuppression, mediated by different cell types such as macrophages and MDSCs [40,41]. The divergent effects of radiation on the rectal cancer TIME will be explained in the following sections, beginning with the initial aspects of the immune response, and followed by subsequent categorization based on specific cell types.

### 2.1. Immune Cell Recruitment, Infiltration, and Cytokine Signaling

Irradiation generally triggers an immunogenic cell death (ICD), a process characterized by the release of antigens, cytokines and adjuvant components, driving adaptive immune responses in immunocompetent hosts [42,43,44,45]. The adjuvant components, also termed as damage-associated molecular patterns (DAMPs), represent danger signals required for the recruitment of antigen-presenting cells (APCs) [44,45]. A correlation between the TME and the tendency of cancer cells to undergo immunogenic cell death, and therefore priming of an anti-tumor response, has been described in different cancer entities [45,46,47]. A critical step for immune surveillance is the relocation of immune cells into the tumor site. The infiltration of innate and adaptive immune cells into the TME is known to create a dynamic network of interactions and to play an important role in regulating tumor progression [48,49,50]. Radiation has been reported to alter immune cell infiltration in different cancers, including rectal cancer, through three main mechanisms [51]: (i) vascular remodeling [52,53], (ii) increase in adhesion molecules [53,54], and (iii) chemokine induction [55,56].

Upon RT or CRT of rectal cancer, the proliferation of tumor and endothelial cells (ECs) was observed to decrease, whilst the expression of adhesion molecules increased, leading to enhanced infiltration of leukocytes [53]. Interestingly, in human CRC tissue, the number of proliferating ECs was found to correlate inversely with infiltration of leukocytes and was identified as a negative prognostic factor [57]. Furthermore, a decline in tumor microvessel density after radiation could be seen in rectal cancer but not in normal rectal mucosa [53]. Therefore, in addition to its direct cytotoxic effects, radiation may facilitate immune cell invasion and contribute to the activation of a local anti-tumor immune response in rectal cancer via modifications in the tumor and its adjacent vascular bed [53].

Lee et al. evaluated the systemic immune response after nCRT for rectal cancer and reported a decrease in peripheral cytokines associated with tumor progression and resistance, one month after CRT [58]. C-C motif chemokine ligand (CCL)2 and CCL3, which have been associated with CRC progression [59,60,61], presented a significant decline one month post-CRT [58]. In addition, CCL2 was reported to be involved in the recruitment of immunosuppressive immune cells [61,62]. The C-X-C motif chemokine ligand (CXCL)12, also known as stromal cell-derived factor-1 alpha (SDF-1α), and latency-associated peptide (LAP) levels, used to evaluate transforming growth factor-ß (TGF-ß) levels, showed an initial decrease upon CRT application followed by an increase during the first month after CRT termination and decline afterwards [58]. CXCL12 [63] and TGF-ß [64,65] have both been implicated in processes contributing to an immunosuppressive microenvironment in CRC, including recruitment of immunosuppressive immune cells. In line with these findings, another study comparing nRT-treated (5 × 5 Gy) and non-irradiated surgically resected rectal cancer samples found lower active TGF-β1 in tumor samples of the irradiated group [66]. However, Yasui et al. observed opposing results: when comparing rectal cancer patients who received nCRT (50.4–66 Gy) to patients who did not, TGF-ß was found upregulated (*TGFB3*) and immuno-fluorescent staining for TGF-β1 revealed that it was derived by cancer cells following nCRT [67]. Analysis of further marker genes, such as signal transducer and activator of transcription 3 (*STAT3*) and SRY-box transcription factor 2 (*SOX2*), revealed induction of an immunosuppressive TIME status [67]. Possible reasons for this discrepancy, that should be further investigated in rectal cancer, are dosage differences as well as the influence of CT in a CRT regimen compared to RT only.

Additionally, Lee et al. [58] observed a decrease in interleukin (IL)-12 serum levels upon CRT, which started to recover one month after termination of CRT. Notably, IL-12 is a known promoter of anti-cancer immunity, as shown in multiple preclinical models for different cancer entities including CRC [68,69,70]. Moreover, previous research has indicated that IL-12 can potently activate cluster of differentiation (CD)8^+^ T and natural killer (NK) cells and stimulate interferon (IFN)-γ production [71].

IFNs type I (IFN-α and IFN-β) and type II (IFN-γ) play a key role in the coordination of anti-tumor immunity [72]. In the TME of CRC, IFN-γ is produced by a variety of cell types, with T cells and NK cells being the main sources [73]. As most inflammatory cytokines, IFN-γ exhibits pleiotropic effects, which can be influenced by the diverse TME [74,75]. In CRC, its anti-tumor properties include the upregulation of the major histocompatibility complex (MHC) class II receptor HLA-DR (human leukocyte antigen DR) [76] and apoptosis promotion in stem-like cancer cells [77]. To date, most available studies have primarily focused on IFN modulatory effects on CRC, whilst studies specifically investigating its influence on rectal cancer remain scarce. Nevertheless, pathway analysis of upregulated genes comparing pre- and post-(C)RT rectal cancer samples of good responders, revealed a significant upregulation of IFN-γ response, showing its importance in mediating an efficient anti-tumor immune reaction after RT [78]. Furthermore, immune biomarker scores of the IFN-γ signature [36] and IFN-γ expression measured via immunohistochemistry (IHC) [79] were found increased in LARC patient specimens following nCRT.

Plasmacytoid dendritic cells (pDCs) are a subset of DCs specialized in producing type I IFNs [80,81]. It was observed that pre-CRT rectal cancer contained just few IFN-α-expressing pDCs, whilst this proportion was increased after CRT, leading to the conclusion that CRT could enhance IFN-α expression in rectal cancer [82]. In established mouse models using the poorly immunogenic MC38 colon cancer cell line, it was found that the IFN-α gene transduction could reduce the cancer cell’s tumorigenicity and evoke a potent anti-tumor immune response with increased infiltration of cytotoxic T lymphocytes (CTLs) [83,84]. These findings highlight the importance of specific investigation whether radiation-triggered elevation of IFN-α in rectal cancer could contribute to an effective RT response by enhancing CTL infiltration and function to boost anti-tumor immunity. CRT further induced the expression of CXCL10 and CCL4 in pDCs infiltrating rectal cancer [82], chemokines that have been associated with T-cell migration [85], implying that pDCs might play a role in T cell trafficking to rectal tumors after CRT.

To conclude, RT and CRT have the potential to enhance the crucial initial steps of the anti-tumor immune response: recruitment and infiltration of immune cells. Through danger signal release upon radiation, immune cells are attracted to the tumor site and their infiltration might be facilitated through modifications of the tumor vasculature and cytokine levels. However, it is important to note that literature on cytokine alterations after rectal cancer RT is scarce, highlighting the need for further investigation in this field. On the other hand, it is crucial to remember that radiation is physically detrimental towards cells, including the cells of the TIME. Therefore, the depletion of immune cells collectively must be considered as important process of immunosuppression upon (C)RT [86]. This effect influences the composition of the TIME since lymphocytes are more radiosensitive than other immune cells, such as macrophages or granulocytes [87,88]. Interestingly, among lymphocytes, regulatory T cells (Tregs), which are associated with immune evasion by tumors, appear to be the most radioresistant cell type [89,90], strongly suggesting that immunosuppressive elements are less affected by the process of physical destruction within the TIME.

Therefore, radiotherapy exerts both: immunostimulation and immunosuppression in terms of general immune cell infiltration and differentiation. Therapeutically, these effects could potentially be enhanced or inhibited. (Pre)clinical trials have evaluated the modification of IFN pathways in addition to RT [91,92,93]. However, the results remain controversial and raise the question of potential toxicity [93].

### 2.2. Treatment-Induced Effects on Innate Immunity

#### 2.2.1. Tumor-Associated Macrophages 

Tumor-associated macrophages (TAMs) are a main component of the TIME and known to play a role in tumor growth, progression, and metastasis [94,95,96]. Although their infiltration in CRC is initially associated with tumor suppression by orchestrating the anti-tumor immune response in early stages [97], overall it remains controversial which effect they have on the tumor [98,99]. They exhibit remarkable functional diversity as they perform central tasks in phagocytosis and antigen presentation and show a high level of plasticity under immunomodulatory conditions [100,101]. Their ample heterogeneity has been shown to be influenced by the TME in CRC [102]. To account for their diversity, their phenotype has been classified in M1 and M2 macrophages based on their surface markers, secretory profiles, and functions [103,104,105]. This initial classification mirrored the basic T helper (Th)1 cell and Th2 cell polarization profiling [103]. Whilst the M1 phenotype is characterized as proinflammatory and tumoricidal, the M2 type is associated with repair, tuning of the immune response and tumor progression [104,105,106]. However, it has become increasingly clear that there are several subsets of TAMs, highlighting their great heterogeneity and calling for redefinition of the traditional classification [107,108]. Additionally, their plasticity, allowing the reprogramming of their phenotype according to environmental settings, is increasingly acknowledged [100,101].

Using flow cytometry after ex vivo irradiation of primary rectal cancer tissue samples, Stary et al. reported that low-dose RT resulted in a change in TAM polarization from the M2-like to the pro-inflammatory M1-like phenotype [109]. Furthermore, the phagocytic capacity of irradiated TAMs was found to be enhanced, going in line with downregulation of programmed cell death receptor 1 (PD-1) [109], a well-known inhibitor of phagocytosis [110]. Additionally, when these findings were translated in vivo by analyzing irradiated patient-derived rectal cancer samples, Stary et al. observed an increased M1/M2 ratio upon RT. The study furthermore presented results indicating that this effect might be mediated by extracellular vesicles released from irradiated tumor cells containing factors driving the phenotypic switch, but the precise underlying molecular pathways require further investigation [109].

Gene expression profiling performed by Wilkins et al., comparing pre-(C)RT vs. post-(C)RT rectal cancer samples, showed longitudinal upregulation of *CYBB* and *CD68*, genes reflecting increased macrophage populations, in good responders, unlike poor responders [78]. The authors noted that at pre-treatment baseline, poor responders demonstrated an intrinsic inflammatory phenotype with upregulation of *CD163*, a marker for the M2-like macrophage subtype. It was suggested that a favorable response to (C)RT in rectal cancer is attributed to a transition from an immunologically “cold” to “hot” state [78]. To conclude, under specific conditions and depending on the TME, TAMs might be an important element of the anti-tumor response after rectal cancer RT.

Strongly contrasting these findings, Cho et al. observed that in LARC patient samples after nCRT, TAMs mainly changed their phenotype to M2, suggesting that they suppress the anti-tumor immune reaction [31]. These findings are in line with results by Yasui et al., who showed more M2 macrophage infiltration following nCRT compared to tumors pre-nCRT [67]. Moreover, they observed an elevation in TGF-ß expression by the tumor cells [67], which is known to attract immunosuppressive TAMs [111]. Remarkably, these results contradict the findings by Stary et al., who reported a differentiation of TAMs to the M1 phenotype following irradiation [109]. The main reason for this discrepancy could be differentially applied forms of RT: Stary et al. used material from patients who received SCRT at a dosage of 2 × 2.5 Gy per day over a course of five days [109]. In the other two studies, however, all applied RT regimens were performed with at least 50 Gy. Further, the neoadjuvant treatment of the latter included chemotherapeutics. Essentially, this discrepancy reflects that RT can elicit opposing effects on the TIME, which may depend on the applied dosage or its fractionation.

Moreover, the classification of TAMs into M1- and M2-polarized macrophages is an insufficient approach to reflect the TIME reality. Some macrophages can express cell markers which occur in both subtypes without allowing distinction, such as CD163 which originally characterizes M2 polarization but can also be found in more M1-like macrophages [112]. This underlines the great heterogeneity of TAM subtypes and their intermediate forms, which can be found in the rectal cancer TME, and therefore contributes to inconsistent findings regarding their function and distribution [113,114]. Pinto et al. evaluated how human-derived macrophages maintained in vitro would react to RT [115]. The macrophages did not only stay viable but also showed a pro-inflammatory polarization: anti-inflammatory gene markers (*CD163*, *MRC1*, *VCAN*) as well as the immunosuppressive cytokine IL-10 were decreased, whilst pro-inflammatory markers (*CD80*, *CD86*, *HLA-DR*) were increased, which could add to anti-cancer immunity. Moreover, the irradiated macrophages exerted pro-invasive and -angiogenic effects when cultured with a colon cancer cell line [115]. Yet, it remains to be tested whether these results can be translated to rectal cancer and to an in vivo situation.

Thymidine phosphorylase (TP) expression by TAMs could have an influence on progression and treatment of rectal cancer. However, its role is not yet fully understood: on the one hand it promotes CRC growth due to pro-angiogenetic and anti-apoptotic effects on the cancer cells [116]; on the other hand, it is responsible for the catalyzation of the widely used chemotherapeutic 5-fluorouracil into its more active nucleoside form, 5-fluoro-2′deoxyuridine [117]. Interestingly, for rectal cancer patients a significant correlation between high TP levels and poor outcome was found after application of nCRT [118]. Additionally, a study found TP production by CD68^+^ macrophages to be increased after irradiation in samples of rectal cancer patients as well as colon cancer cell lines [119]. Furthermore, monocyte chemoattractant protein-1 (MCP-1/CCL2) was also increasingly expressed by the tumor cells one week after irradiation, indicating a higher recruitment of TAMs into the TME and a cross-talking signaling between cancer cells and TAM recruitment [119]. A clinical trial with rectal cancer patients observed a rise in TP levels following RT, although the cell type responsible for its expression was not described [120].

**Figure 1:**
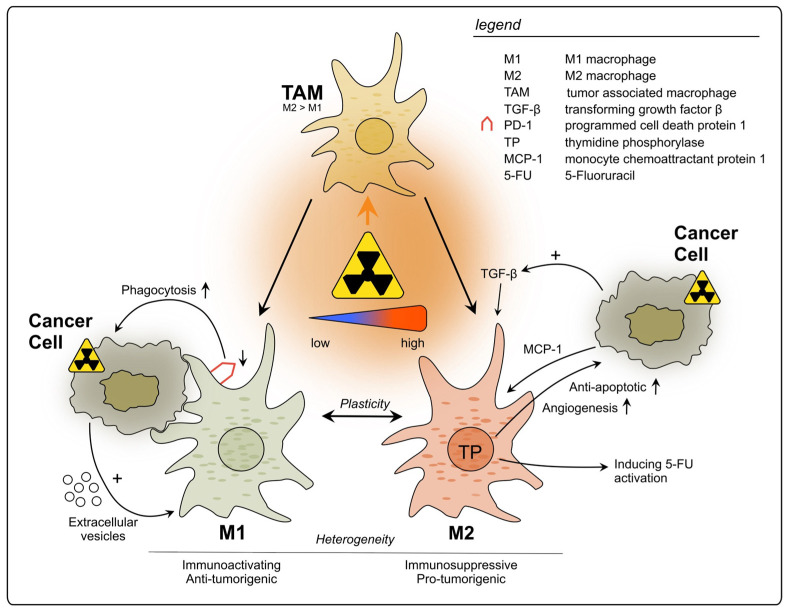
Impact of radiotherapy on tumor-associated macrophages in rectal cancer. TAMs represent a heterogeneous population and exhibit functional diversity. They are classified into two major phenotypes: M1, which is characterized as immune-activating, anti-tumorigenic and M2 with immunosuppressive, pro-tumorigenic properties. Due to high plasticity, TAMs can alter their phenotype depending on the surrounding conditions. Conflicting results have been reported regarding the polarization of TAMs upon rectal cancer RT, with studies describing increase in polarization towards either M1 or M2. This discrepancy may be attributed to differences in the dosage of radiation applied during the treatment.

In summary, TAMs can be triggered by radiation to exert pro-tumorigenic effects, mainly through the production of immunosuppressing factors and cytokines, which can affect not only tumor growth and metastasis, but also its treatment outcome (see Figure 1). Nevertheless, further studies using different RT doses and fractionation are necessary to fully understand their roles upon RT, since some evidence suggests positive effects on TAM-mediated anti-cancer immunity. Additionally, the baseline immune status of tumors should be acknowledged when evaluating RT immune effects. Therefore, it is crucial to further evaluate which RT conditions are least likely to activate tumor-promoting TAMs and hinder anti-tumor immunity.

#### 2.2.2. Neutrophils

Next to their role as essential defenders against microbial infections, neutrophils have gained attention as significant regulators in the context of cancer [121,122]. Similar to macrophages, neutrophils can be polarized into tumor-promoting or tumor-suppressing subtypes depending on the TME [123,124]. High neutrophil blood counts were shown to be associated with worse survival of rectal cancer patients [125]. Furthermore, the neutrophil-to-lymphocyte ratio (NLR) has gained attention in recent years as prognostic marker for rectal cancer [126,127,128], which underlines the important role of neutrophils in anti-tumor immunity. Recently, a meta-analysis revealed that elevated NLR before CRT, but not after CRT, is associated with poorer prognosis [126]. Nonetheless, the prognostic impact of intratumoral neutrophils in rectal cancer remains uncertain [129,130,131,132]. Previous studies have indicated that neutrophils play a role in creating an immunosuppressive TME, making them a potential treatment target [122]. However, opposing results have been reported concerning the response of neutrophils upon RT [122].

During the first two weeks of CRT, a decrease in neutrophils in rectal cancer patients’ blood samples was reported, but numbers increased again one month after termination of CRT [58]. Additionally, it was observed that rectal cancer patients who achieved a pCR demonstrated significantly lower peripheral blood neutrophil counts compared to those who did not achieve pCR two weeks after CRT initiation, but not upon the beginning of the treatment [58]. Another study found a significant decrease in the intratumoral and peritumoral numbers of neutrophils five days after RT of rectal cancer [133]. These limited findings suggest that RT may potentially decrease the immunosuppressive and tumor-promoting properties of neutrophils in rectal cancer by reducing their numbers. However, studies in mouse models of other tumor entities reported a peak of neutrophil levels after 24 h with a subsequent decline, highlighting their role as initial innate responders [134,135]. Those studies observed that RT could enhance cytokine release leading to increased neutrophil infiltration and to promote a switch towards an anti-tumor phenotype upon RT [134,135]. Reactive oxygen species were found to play a central role mediating the radiosensitizing, tumor-suppressing activity of irradiated neutrophils [134,135]. Therefore, research should be conducted in rectal cancer after shorter time intervals since neutrophil levels undergo dynamic changes.

However, some studies suggest an unfavorable influence in rectal cancer [136,137]. In a retrospective analysis performed with 73 LARC patients, circulating neutrophils remained stable in numbers during CRT [136]. Furthermore, a high neutrophil blood count post-nCRT correlated with an unfavorable outcome, which was associated with their suppressive effects on T cells [136]. This effect is mediated through the production of NOS and/or the release of arginase I upon their active degranulation or death [138,139,140].

On the contrary, in another study where the granulocyte blood count of LARC patients was evaluated after nCRT, a significantly lower number of these cells was found when compared to patients who did not undergo preoperative therapy [86]. This could be shown until two days after surgery 4–6 weeks after the nCRT and was interpreted as a sign of immune dysfunction and associated with postoperative complications. However, the granulocyte counts strongly increased after the surgery in both groups, probably related to the well described systemic inflammatory response post-surgical interventions [86].

Yang et al. evaluated in their study whether the NLR could be associated with the risk for distant metastasis in LARC patients [137]. They found not only that a high NLR was indicative for distant metastasis and a poorer survival but also that the NLR rise depended on the used RT modalities, an effect which could be attributed to the exposure of the bone marrow to low-dose radiation during intensity-modulated RT leading to leukocytopenia and therefore triggering a NLR rise. Furthermore, LCRT is believed to lead to a higher NLR than SCRT [137].

Summing up, the effects of rectal cancer RT on neutrophils are controversial and their clinical implications are still poorly evaluated, even though their prognostic relevance is well known. The limited availability of literature makes it currently impossible to provide a definitive assessment of the functional characterization of neutrophils upon rectal cancer (C)RT. Well-defined experimental setups and clinical studies will be crucial the development of treatment modalities that influence neutrophil phenotypes in a manner that favors patients’ outcome.

#### 2.2.3. Myeloid-Derived Suppressor Cells

Myeloid-derived suppressor cells (MDSCs), firstly described as “natural suppressor cells” in 1978 [141], encompass a heterogeneous group of immature myeloid cells that play an important immunosuppressing role in the TME [142,143]. They can be divided in two main categories: polymorphonuclear (PMN-MDSCs) and monocytic (M-MDSCs) [144,145,146]. However, due to broad ambiguity regarding clearer definitions of this cell population, the general term MDSC is still widely used [147]. In cancer, they generally exert immunosuppressive effects via multiple direct and indirect mechanisms, including suppression of T cell activation, Treg development and modulation of cytokine production by macrophages [142,143,148]. Accordingly, in rectal cancer MDSCs are primarily known to contribute to immunosuppression and treatment resistance [149,150].

In a LARC cohort of 25 patients, pre- and post-nCRT intratumoral MDSC levels were found significantly higher in non-responders, implying that higher intratumoral MDSCs before nCRT of rectal cancer correlate with non-responsiveness [149]. In line with this finding, high MDSC infiltration in surgical LARC specimens post-nCRT was associated with worst prognosis [150]. Teng et al. found no change in the expression of MDSCs due to CRT in samples of rectal cancer patients, but they observed that those patients who presented lower levels of MDSCs prior to therapy had a better outcome than those showing higher MDSC numbers [34]. Acknowledging the impact of the high individuality of the TIME, measuring MDSC levels could be used to implement personalized treatment strategies by identifying patients who are likely to benefit from CRT and those with poorer prognosis who may require additional interventions [149,150].

Concerning the exact mechanism involved in treatment resistance, specific literature on rectal cancer is lacking. Blood samples from 41 CRC patients were compared to samples from eight healthy donors and a significant expansion of M-MDSCs was found, which exerted immunosuppressive properties in a mixed leukocyte reaction (MLR) assay where the MDSCs were mixed with DCs as well as T cells [151]. T cell proliferation was evaluated after five days by measurement of ^3^H-thymidine incorporation and shown to be significantly reduced compared to a MLR with CD14^+^/HLA-DR^hi^ monocytes. Moreover, patients who received an anti-cancer treatment (surgery, CT, RT, targeted therapy, or a combination of these methods) showed higher levels of M-MDSCs expressing CD38 when compared to treatment-naïve CRC patients. The authors proposed therefore, that CD38 could be a promising target for immunotherapy. However, there are limitations to this study since all treatments were pooled together as a single group and the exact number of rectal cancer patients within the cohort was not defined (“CRC patients”) [151].

The amino acid L-arginine is of relevance for anti-tumor-immunity: classically activated macrophages can convert it into nitric oxide which is able to enhance tumor reoxygenation under hypoxic conditions and therefore functions as a radiosensitizer through ROS-mediated DNA damage [152]. Furthermore, T cell proliferation and M1 macrophage activation are stimulated by L-arginine [140]. It has been proposed that one essential mechanism of MDSC-mediated radioresistance in CRC patients is the depletion of L-arginine via the enzyme arginase-1 [153,154]. Leonard et al. showed that MDSCs exerted these properties in a mouse model using CT26 colon cancer cells in which they impeded activation of anti-tumorigenic macrophages via L-arginine consumption, and thereby observed a radioprotective effect [155]. Furthermore, they observed high counts of arginase-1-producing MDSCs as well as neutrophils in the blood of 235 LARC patients compared to 15 healthy donors. It was not evaluated whether RT would change the number of MDSCs in the patients; however, the authors strongly suggested that MDSCs would impair RT in LARC patients via inhibition of the above-mentioned mechanisms [155].

In a LARC cohort of 25 patients, it was observed that intratumoral MDSCs were effectively reduced by nCRT in responders and non-responders; however, the exact mechanism was not specified [149]. Moreover, a subset of immature myeloid cells characterized as HLA-DR^−^/CD33^+^/CD16^−^/CD11b^+^ was significantly less pronounced in tumors of good responders compared to non-responders. However, blood levels of this subset were strongly increased after 6–8 weeks in responders and non-responders, shortly before surgery. These cells were experimentally found to have a strong suppressive effect on TILs, compared to other myeloid cell subsets [149], probably due to direct contact-mediated inhibition of T cells [156], but not exclusively. Even though not having direct immunosuppressive abilities, by possible colonization of the tumor, circulating MDSC subsets could exert tumor-promoting effects post-treatment.

A trend towards MDSC reduction was also observed in colon cancer mouse models, where single high-dose radiation decreased MDSCs 14 days after irradiation in a mechanism depending on CD8^+^ cross-priming DCs, IFN-γ secretion and CD40L-expressing CD4^+^ T cells [157]. Shortly after RT, increased infiltration of CD8^+^ T cells was found to reverse the immunosuppressive TIME state via IFN-γ production, which was suggested to regulate MDSC infiltration and survival, leading to lasting remission [157]. (C)RT of rectal cancer was shown to induce infiltration of CD8^+^ and CD4^+^ T cells [31,32,34], DCs [31] as well as IFN-γ upregulation [36,78,79]. Therefore, these effects could be further investigated as triggers of MDSC elimination in response to rectal cancer radiotherapy.

MDSCs are primarily known to contribute to immunosuppression and treatment resistance in rectal cancer [149,150]. Furthermore, novel treatment approaches should be considered to alleviate their contribution to radioresistance, such as immune population-specific depletion, induction of cell maturation or inhibition of their recruitment to the tumor site, as reviewed elsewhere [158]. However, downregulation of MDSC infiltration could be a possible immune-activating effect of (C)RT in rectal cancer. Further research addressing different treatment regimens will be crucial for the full understanding of this scenario.

#### 2.2.4. Natural Killer Cells

With their potent cytolytic function, NK cells are important players in anti-tumor immunity [159]. When compared to normal tissue, lower NK cell counts were observed in rectal cancer [160]. NK cells were shown to engage in a crosstalk with CD8^+^ T cells in CRC, enhancing the anti-tumor immune response and supporting prolonged survival [161]. Radiation was reported to promote NK cell activity and migration to the tumor site in different tumor entities, depending on the applied dosage and surrounding TIME [162,163].

In LARC, an increase in the number of NK cells and in the expression of NK cell-associated genes was found after long-course CRT, which correlated with better OS [164]. Furthermore, low NK cell activity was identified as an important factor for the development of metastasis in stage III rectal cancer after surgery [165]. Yet, in a group of rectal cancer patients, nCRT was reported to impair NK cell activity, thus possibly facilitating metastasis, which represents an additional reason for the combination of CRT with immunotherapy [165]. This study underlined the heterogeneity of the TIME, leading to highly individual (C)RT responses that make it difficult to predict treatment efficiency. NK cells are influenced by a variety of immune cells, such as TAMs, DCs and Tregs so their activity depends on the TIME composition [162,166]. Furthermore, the impact of radiation on NK cell function in different tumor entities depends on the intensity: whilst low-dose ionizing irradiation tends to activate NK cells, high-dose irradiation is more prone to cause partial impairment of NK cell functions [162,167].

To conclude, further specific research considering different radiation regimens is needed to clarify the role of NK cells in the rectal cancer TIME. Nevertheless, NK cells represent a promising target to enhance anti-tumor immunity and are under investigation as strategic effectors of novel immunotherapeutic approaches, such as chimeric antigen receptor-transduced NK (CAR-NK) cells [168].

#### 2.2.5. Dendritic Cells

DCs represent a system of specialized APCs playing a key role in the initiation and regulation of immune responses [169,170,171]. Whilst immature DCs primarily capture and process antigens, mature DCs are capable of initiating a potent immune response by priming of T cells, which in turn interact with other immune cells, such as B cells and macrophages [169,171]. Regarding rectal cancer, DC density was reported to increase after CRT, and higher levels of these cells at the pre-treated LARC tumor site were found to be associated with positive response to nCRT [31]. Furthermore, correlations between stromal DCs and CTLs were reported, suggesting a CTLs cross-priming by DCs, enhancing the anti-tumor immune reaction in rectal cancer [172]. 

6-sulfo LacNAc-expressing monocytes (slanMos) represent a subset of monocytes that produce proinflammatory cytokines, mediate tumor-directed cytotoxicity and can acquire DC functions [173,174]. When investigating the impact of nCRT of rectal cancer on the phenotype of infiltrating slanMos and pDCs, Wagner et al. found an increase in the amount of slanMos secreting nitric oxide synthase (iNOS) or TNF-α and of pDCs locally expressing IFN-α [82]. Additionally, a greater amount of the maturation marker CD83 was observed on pDCs in rectal cancer post-CRT [82].

Therefore, CRT could support anti-tumor immune response not only by increasing the amount of DCs, but also via phenotypical alterations, converting them from an immature status into mature cells, affecting cytokine secretion and cross-activation of other immune cells [82]. Due to the central role of DCs within the interconnected TIME [172,175], these alterations in response to RT have the potential to positively influence multiple processes of anti-tumor activity.

### 2.3. Treatment-Induced Effects on Adaptive Immunity

As part of the immune response to malignancies, tumor-infiltrating lymphocytes (TILs) have been reported to predict prognosis in rectal cancer [33,35,176], associating high levels with a positive outcome. CRT was shown to alter the number of TILs in rectal cancer in several studies with trends varying amongst cell types [31,33,35]. The total lymphocyte count was observed to increase within the TIME of LARC patient samples 4–6 weeks after CRT [160].

#### 2.3.1. Cytotoxic T Lymphocytes and T Helper Cells

In rectal cancer, the population of stromal CTLs was observed to be significantly lower in patients that received RT within two weeks before surgery, compared to patients who received RT followed by a 6–8 week waiting period prior to surgery, suggesting that a CTL repopulation takes place [172]. Another study by Mezheyeuski et al. found that not only CTLs but also other immune cells, e.g., CD8^+^ Tregs, M2 macrophages and pDCs decreased in rectal cancer tissue after SCRT followed by immediate surgery within 3 weeks [27]. However, the group that underwent delayed surgery (later than 3 weeks after RT or CRT) demonstrated an increase in these cells and taken together, the immune infiltrate levels resembled those of tumors unexposed to radiation [27]. This initial decline of immune cells followed by their subsequent recovery was also observed in peripheral blood samples after CRT for LARC [58]. Lymphocyte counts were reported to decrease within the first month after CRT onset, followed by an increase one month after termination of the treatment [58]. A continuous decline was only observed for lymphocytes and not in neutrophils or monocytes under CRT [58]. This reaction goes in line with the high radiosensitivity of lymphocytes compared to other peripheral blood cells [87,88]. Possible explanations for the initial decrease were the suppressing impact of RT on the pelvic bone marrow and the hematologic toxicity of the capecitabine-based CT [58,177,178]. 

Providing a more detailed view on alterations in TILs upon irradiation, CD3^+^ T cell density was reported increased after CRT [31,33] and RT [33] and high values were a positive prognostic factor contributing to immune activation after CRT in rectal cancer [31,33]. Nevertheless, another study found CD8^+^ and CD3^+^ T cells to be decreased after nCRT of rectal cancer, implying an immunosuppressive therapy effect [179]. These findings highlight the complexity of the immune response after rectal cancer RT, which is influenced by many factors and exhibits inter-patient variability [78].

CD8^+^ CTLs were reported as the predominant population amongst the systemically increased lymphocytes after radiotherapy [58]. They are a crucial component of the anti-tumor immune response due to their ability to kill cancer cells after recognition of antigens presented to them [180,181]. CD8^+^ cells were reported increased in multiple studies of rectal cancer after RT [33] and CRT [31,32,33,34,35,36,82] and a high number before treatment was associated with good response to CRT and better prognosis [31,33,34,35]. Interestingly, a study reported that CD8^+^ TILs were not increased after nCT in rectal cancer, but after nCRT, which led to the conclusion that radiation might induce a stronger anti-tumor immune response than CT [32]. Different studies found an increase in CD8^+^ T cells expressing granzyme B, a cytotoxic effector molecule [182,183], in nCRT-treated rectal cancer compared to untreated tumors, suggesting enhanced anti-tumor immunity [82,179]. The activity of CD8^+^ T cells in anti-tumor response is also strongly influenced by CD4^+^ T cells [184,185], that have also been found increased after CRT in rectal cancer biopsies [34]. APCs present antigens on the MHC II receptor to CD4^+^ cells that enable them to then activate CD8^+^ T cells [184,185]. Thus, CRT not only influences lymphocyte numbers but also their activity.

However, Graham Martínez et al. reported increased CTLs and lower Th cells in human rectal cancer samples after nCRT in comparison to nCT [172]. Moreover, when comparing RT and CRT with a similar interval and RT regimen, they found less stromal Th cells in CRT-treated patients and a trend towards lower B cells and Tregs as well as more tumor-infiltrating DCs. These findings suggested that a synergistic effect of RT and CT might induce CTL proliferation and DC infiltration whilst depleting other stromal immune cells [172].

The immune reaction after treatment including RT appears to be more homogeneous compared to no treatment or CT, and Th cells were identified as key players in this variance [172]. A possible explanation, given by Graham Martínez et al., could be the coordinated response following the refractory period after RT. Therefore, and considering the more local effect of RT compared to CT, the authors concluded that the TME might be more affected by RT than CT [172]. This supports the use of systemic immunotherapies, such as immune checkpoint inhibitors, which have gained attention for their ability to enhance the immune-stimulating alterations induced by RT [40,186].

In addition to its local impact, RT has also been shown to have systemic activating immunologic effects. As already mentioned, radiation kills local tumor cells via ICD, thereby recruiting APCs that induce B-cell- and T-cell-mediated immune responses [42,43,44]. The resulting immune priming might tackle cancer cells outside of the irradiated area, a mechanism known as “abscopal effect”, which is why RT has been described as in situ vaccine [42,187]. It was reported that an oxaliplatin induction followed by a reduced oxaliplatin dose plus full-intensity irradiation of rectal cancer could induce and maintain ICD, which seemed to protect against metastatic progression [188]. The amount of ICD increase was negatively associated with metastatic failure risk, indicating systemic immune effects triggered by CRT [188].

**Figure 2:**
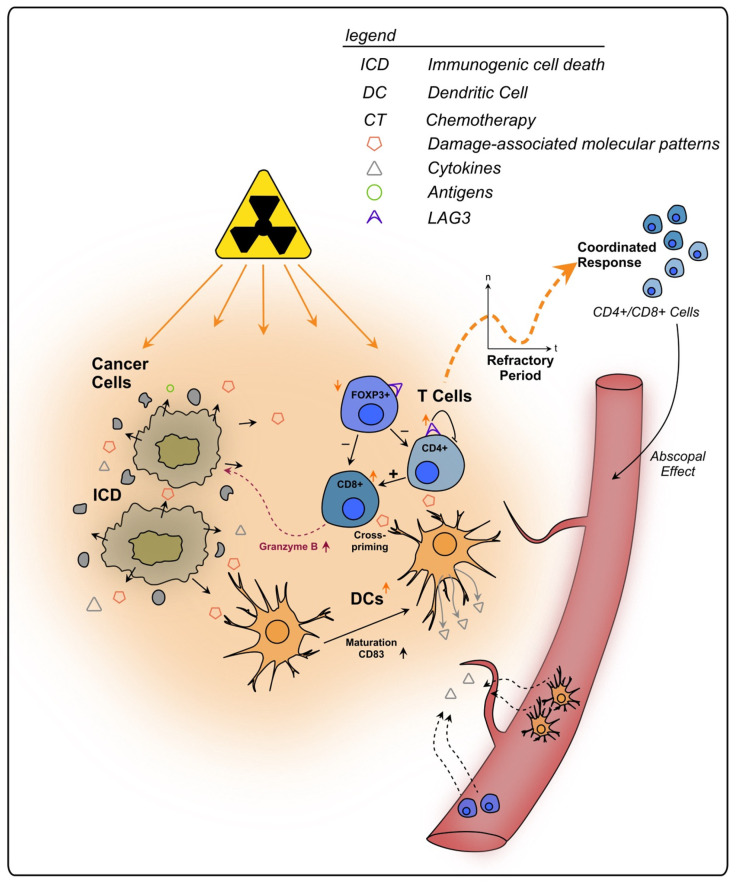
Effects of radiotherapy on tumor infiltrating T lymphocytes in rectal cancer. Irradiated cancer cells undergo immunogenic cell death, triggering the release of DAMPs and cytokines. Subsequently, immune cells are recruited from the bloodstream to the tumor site. Following an initial refractory period immediately after RT, lymphocytes increase within the TIME in a coordinated response. APCs, such as DCs, also show an increase in number and exhibit enhanced maturation markers upon RT. They present tumor antigens to T cells, priming them towards the tumor. CD8^+^ T cells can directly kill cancer cells, e.g., via granzyme B, and are further activated by CD4^+^ T cells. Additionally, T cells primed towards the tumor are enabled to kill cancer cells outside of the tumor site, a process termed as abscopal effect. RT stimulates the expression of the regulatory cell surface receptor LAG-3 on T cells which are thereby impaired in their anti-tumorigenic functions.

To conclude, lymphocytes play an integral role in the immune response of rectal cancer following RT and CRT (see Figure 2). Through its severe local effects, RT might influence the TIME stronger than CT, whilst also triggering systemic effects, such as immune priming outside of the irradiated area (abscopal effect). The initial decrease in immune cells and their re-emergence after irradiation should be considered when planning a combination therapy, e.g., with checkpoint inhibitors and surgery, since differences in immune activation might change the impact of those interventions. A better understanding of this dynamic is necessary for a (more) precise clinical approach against rectal cancer.

#### 2.3.2. Regulatory T Lymphocytes

The transcription factor forkhead box P3 (FOXP3) is a specific marker for CD4^+^CD25^+^ Tregs and programs their development and function [189,190,191]. Tregs are suppressor T cells that play a crucial role in the prevention of autoimmunity [192,193] and can crucially impair anti-cancer immunity due to their ability to actively inhibit the activation and expansion of effector T cells [194,195]. The density of FOXP3^+^ TILs was reported to decrease in rectal cancer after RT [196] and CRT [31].

McCoy et al. reported that low stromal Foxp3^+^ Treg density in LARC after CRT of 50.4 Gy in 28 fractions over 5 weeks correlated with pCR and better long-term prognosis [197]. They concluded that Tregs in the rectal cancer TIME might inhibit the anti-cancer immune response following nCRT and therefore represent a therapeutic target [197]. In line with this, Cho et al. found that strong increase in Tregs in LARC after nCRT in a median dose of 50 Gy was associated with poor disease-free survival [31]. Furthermore, Napolitano et al. found a decrease in circulating Treg numbers, together with MDSCs, following SCRT of 13 LARC patients, not only during, but also five and eight weeks after the treatment [198]. In poor responders according to tumor regression, Treg levels increased, and subpopulations expressing the immunosuppressive markers CTLA-4 and PD-1 infiltrated the tumors of those patients [198].

Intriguing, it was also observed that high density of tumor-infiltrating Tregs was associated with higher levels of CD3^+^ T cells, CD8^+^ T cells, B cells, APCs, and DCs following nCRT, which might be a sign of increased immunity and emphasizes the importance of interpreting immune cell changes in conjunction with other populations [31]. Interestingly, Mirjolet et al. reported that high Treg infiltration and a significant decrease in the CD8^+^/FoxP3^+^ ratio following nRT of LARC were associated with longer progression-free survival, suggesting the establishment of an immunological balance between CD8^+^ T cells and Tregs after radiation [196]. Furthermore, they observed a significant difference in the effect of RT depending on the administration schedule with a higher CD8^+^/FoxP3^+^ ratio after LCRT compared to SCRT [196].

To sum up, immunosuppression via Treg activity may occur in response to (C)RT of rectal cancer, especially in patients who are regarded as poor responders. However, results differ, and RT was also shown to have the potential to suppress Treg numbers and activity (see Figure 2). Therefore, no clear conclusion can be drawn concerning their prognostic impact. Increased levels might also represent an increase in immune activation. Additional research is needed to elucidate the role of Tregs in the rectal cancer TIME upon (C)RT. Nevertheless, depletion of Tregs in addition to RT might pose a promising therapeutical approach, as suggested by CRC animal models’ experiments [199,200,201]. To date, to the best of our knowledge, no (pre)clinical trials addressing therapeutic Treg depletion in combination with (C)RT in rectal cancer have been performed. 

#### 2.3.3. LAG-3^+^ T Cells

Lymphocyte-activating gene 3 (LAG-3) describes a cell surface receptor expressed by T cells, which serves as an inhibitory checkpoint regulator during the interaction with MHC II molecules to prevent autoimmunity [202]. In CRC patients LAG-3-positive Tregs produce IL-10 as wells as TGF-ß1 and are believed to be strongly activated and therefore directly involved in tumor immune evasion, due to direct contact inhibition of other T cells [203]. According to Tayshetye et al., LAG-3 was increasingly expressed post-nCRT in samples from rectal cancer patients indicating that after RT the receptor triggers tumor immune escape via inhibition of tumor-primed T effector cells [204]. This goes in line with another report which observed a LAG-3 rise in IHC analysis of irradiated rectal cancer samples (see Figure 2). Notably, they have also found that the LAG-3 expression was stronger after SCRT than after LCRT [205].

Therefore, combination therapy with RT and a LAG-3 inhibitor is an interesting approach to block immune evasion mediated by this mechanism. First attempts have been made in CRC patients and promising results are reported [206]. However, rectal cancer-specific research on this matter is still required.

#### 2.3.4. B Lymphocytes

B cells, next to their most recognized role as antibody-producing cells, present antigens, co-activate T cells and secrete cytokines [207,208]. As a heterogeneous component of the TIME, their role remains controversial: tumor-promoting as well as anti-tumor effects depending on microenvironmental conditions have been reported [209,210]. A positive prognostic role of tumor-infiltrating CD20^+^ B lymphocytes was described for non-irradiated CRC but not for irradiated rectal cancer and a cooperative effect of CD20^+^ B lymphocytes and CTLs was suggested [211]. Literature on the effect of RT on B cells is scarce but existing evidence suggests that radiation can influence B cells and result in their enhanced infiltration into tumors [212]. A recent molecular analysis of pre-treatment LARC biopsies revealed a correlation of B cell-related gene enrichment with good nCRT response, suggesting that B cells might play a role in CRT-induced anti-tumor immune response [213]. The analysis also showed an increase in antigen presentation pathways in good responders, which led to the suggestion that adjacent B cells, stimulated by antigen release upon CRT, might secrete inflammatory cytokines such as IFNs and present neoantigens to CD4^+^ T cells via MHC II, enabling them to cross-prime CD8^+^ T cells [213].

Nevertheless, the mentioned positive effects can be impaired by cell diminishment through radiotherapeutic treatment. Following CRT of rectal cancer, the density of CD20^+^ B cells that was potentially associated with disease-free survival has been reported significantly decreased [31]. This finding implied immunosuppressive effects of RT on B cells [31]. Unfortunately, no underlying molecular mechanisms have been described in the study for the obtained result. Additionally, when evaluating effects of neoadjuvant rectal cancer therapy on the regional lymph nodes surrounding the tumor, Kolotova et al. found that RT inhibited mitosis and differentiation of intranodal B lymphocytes, which was interpreted as a sign of immune dysfunction [214]. Hence, RT of rectal cancer seems to negatively affect anti-tumor immunity mediated by B cells, primarily via decreased cell counts and reduced antigen presentation capabilities.

All in all, B cells are an important component of the TIME, often found neglected in human experimental oncology. Improvement in the ability to discriminate a recruited functional tumor B cell from a bystander one upon CRT would enhance our understanding of the production of tumor-reactive antibodies in rectal cancer. Crucially, this would contribute to the development of new therapeutical approaches, especially in the field of immunotherapy. To date, few attempts have been made to influence B cells in CRC, let alone in rectal cancer specifically. Whilst one study could provide hints that partial B cell depletion might be of potential benefit [215], research remains limited in this field.

### 2.4. Other Therapy-Induced Immunosuppressive Effects

#### PD-L1 and PD-1

PD-L1, a ligand typically expressed on the surface of immune cells such as macrophages and DCs upon their activation, was first described in 1999 by Dong et al. [216]. PD-L1 is able to bind to its receptor PD-1 on the surface of T and B cells, where it exerts crucial effects on the development of immune tolerance and prevention of autoimmunity. Triggering of the PD-1/PD-L1 pathway leads to a dephosphorylation of effector molecules in T and B cell signaling, thereby attenuating the activation of these cells [217]. In rectal cancer, PD-L1 expression is found increased in tumor cells and is inversely associated with survival [150].

Previous reports suggest that rectal cancer therapy has the potential to influence PD-L1 expression on the tumor cells as well as within the TME. Rectal cancer samples analyzed by IHC showed that patients who underwent nCRT expressed significantly more PD-L1^+^ tumor cells than those who only received a surgical resection [218]. Possible mechanisms mediating the PD-L1 upregulation are DNA damage signaling pathways, IFN-γ signaling, the cGAS-STING pathway and epidermal growth factor receptor (EGFR) signaling [219]. Boustani et al. also observed that RT increased PD-L1 expression in cancer cells of LARC patients [37]. Interestingly, the authors found this effect to be long-termed since it could still be observed at the point of surgery, which was in most cases performed six weeks later [37]. In line, paired analysis of 123 pre-CRT biopsies and their corresponding post-CRT resected tissues showed that after nCRT PD-L1 expression in cancer cells was increased. Furthermore, patients with lower CD8^+^ TIL density presented a poorer survival [38]. Remarkably, patients who already had a high PD-L1 score before nCRT showed a diminished TIL rate after the therapy, highlighting the immunosuppressive and tumor-promoting role of PD-L1 [38].

The exact mechanisms through which PD-L1 leads to immune evasion of rectal cancer upon radiation are still under debate; however, it has been suggested that activation of the ATR signaling pathway in rectal cancer cells could play a major role, preventing their phagocytosis by APCs [39]. This effect could subsequently inhibit an immune priming towards the cancer cells and thereby undermine the effect of RT as an in situ tumor vaccine [220], directly changing the abscopal effect [42,187]. Interestingly, results showing no association between PD-L1 expression in cancer cells and a worse disease outcome have also been reported, yet, the increase in PD-L1 expression after CRT was still observed [221,222]. Therefore, although already acknowledged in most recent studies as a negative prognostic marker in rectal cancer, the specific effects that (C)RT exerts on PD-L1 function are still under assessment, especially in cells of the TME, apart from the cancer cells.

Regarding the PD-L1 expression on immune cells, some research groups could remarkably draw a line between high expression and improved survival in rectal cancer [150] (or CRC [223,224]), which could be interpreted as a sign of a “hot tumor” meaning triggering of a strong anti-tumor immune reaction [150]. Boustani et al. were able to show that LARC patients receiving RT who presented a high-to-high PD-L1 expression in cancer cells had the best overall survival compared to other groups, further supporting the theory of the “hot tumor” phenotype [37]. However, results from Lim et al. contradict these findings by correlating a high-to-high PD-L1 score with impaired activation of CD8^+^ T cells and a poor outcome [38]. In this scenario it can be assumed that an immunosuppressive TIME at baseline (pre-treatment) also impairs the T cell activation post-therapy. Overall, patient heterogeneity might be the reason for the discrepancy between the studies.

Interacting with PD-L1, the T cell surface receptor PD-1 plays a similar immune inhibitory role as its ligand [225]. PD-1 expression on TILs in treatment-naïve patients is repeatedly considered a positive prognostic marker in CRC, being a sign of immune activation [226,227,228,229]. Nevertheless, other studies contradict these findings: Bakhrebah et al. positively correlated the expression levels of PD-1 in whole blood samples with the tumor stage of CRC patients [230]. Further studies identified PD-1 as a negative prognostic factor for OS in patients with stage II/III CRC after curative resection [231] or showed an association between a high PD-1/CD8 ratio and poor OS in CRC patients [232]. Therefore, the prognostic significance of PD-1 is debatable. Notably, no literature specifically addressing this topic in the context of rectal cancer could be found.

Previous animals models using colon cancer cell lines (MC38 and CT26) suggested that PD-1 upregulation on T cells following (C)RT has rather unfavorable effects on T cell activation and anti-tumor response [199,233]. In human mucinous rectal cancer patients, the use of nCRT and high PD-1 were positively correlated [234]. Furthermore, in a small study including 13 LARC patients who had received SCRT, it could be found that poor responders had an increased number of CD4^+^/CD25^hi+^/FOXP3^+^/PD-1^+^ Tregs infiltrating the tumors compared to good responders [198]. These results show that the effects of RT on PD-1 are more ambiguous than on PD-L1, possibly due to the fact that PD-1 upregulation is induced by different factors, such as T cell receptor signaling or various cytokines [235]. Moreover, PD-1 is expressed by different immune cell types [236,237,238,239], which are themselves influenced by RT regarding their cell counts or activation status, leading to a high number of variables affecting the expression pattern.

**Figure 3:**
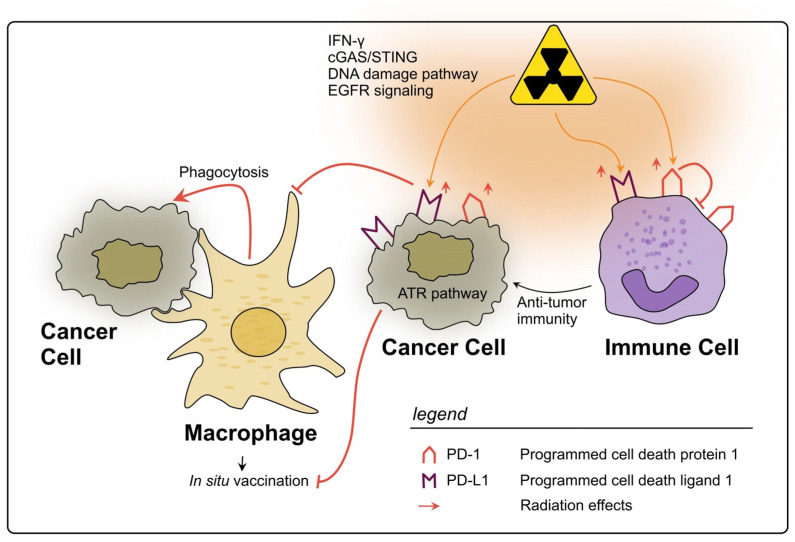
Impact of radiotherapy on the PD-1/PD-L1 axis in rectal cancer. RT may lead to a significant upregulation of PD-L1 on rectal cancer cells through several described pathways. PD-L1 allows cancer cells to evade phagocytosis by macrophages and subsequently impairs the in situ vaccine effect. On immune cells within the TIME PD-L1 is also upregulated by RT and can therefore inhibit their activation. However, high levels of PD-L1 on immune cells could also be a sign of strong immune activation in response to RT. The expression of PD-1 on immune cells can also be upregulated due to RT, thereby inhibiting anti-tumor immunity, e.g., due to impaired T cell activation.

In conclusion, PD-1 and PD-L1 are two biomarkers of the TIME in rectal cancer which are clearly influenced by RT, mostly being upregulated by several cell types, and tending to an unfavorable immunosuppressive therapy response, as a negative side effect of RT (see Figure 3). Still, reflecting the complex interactions within TIME, the prognostic value of PD-(L)1 levels pre- and post-nCRT remains contradictory. However, their targeting in combination with RT is a promising therapeutic approach, which is being tested in clinical trials [240,241].

## 3. Discussion

**Figure 4:**
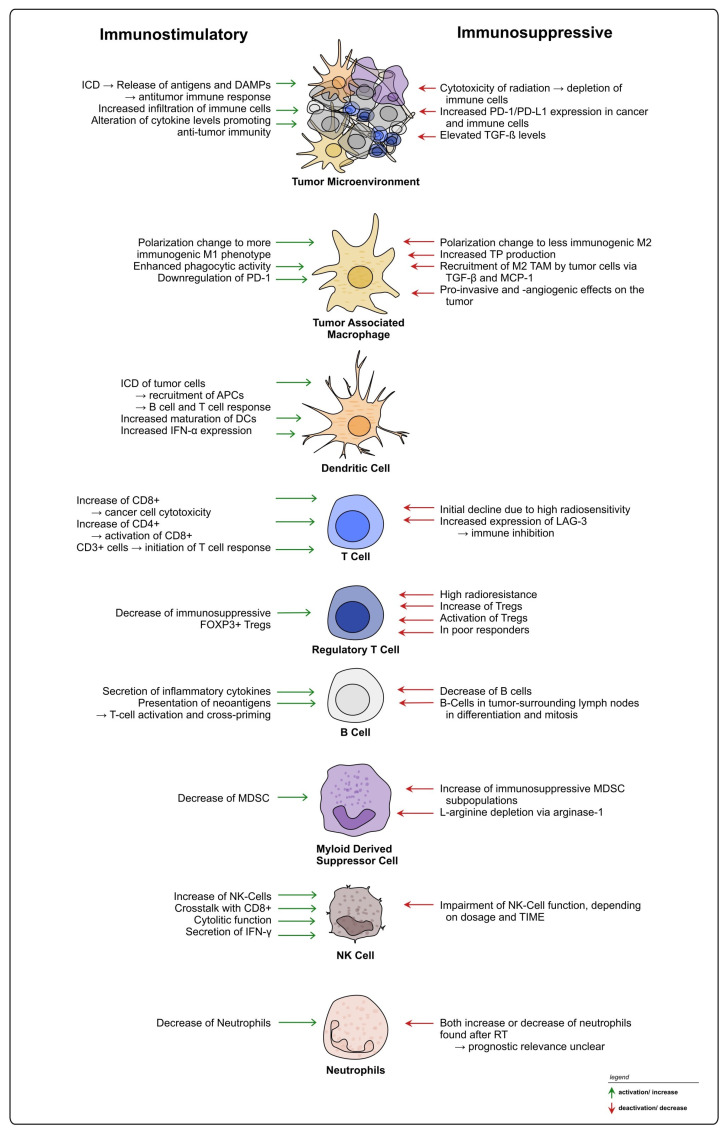
Summary of radiation-induced effects on immune cells in rectal cancer. Overview of possible immunostimulatory and -suppressive effects of radiation on different immune cell types within the TIME of rectal cancer.

It is well accepted that the TIME heavily influences the response to RT and CRT in rectal cancer, exerting both positive and negative effects on treatment and therapy resistance (see Figure 4). TIME heterogeneity represents an obstacle, and currently TIME-mediated RT responses cannot be generalized, with patients exhibiting different outcomes under similar treatment regimes. Moreover, based on the available literature, it is evident that not only treatment-induced changes on the TIME, but also TIME-intrinsic characteristics prior to the intervention should be considered when planning treatments including radiation and potential combinational therapies for rectal cancer [78]. The identification and establishment of additional new biomarkers within the tumor and the TME could be used to provide patients with personalized treatment strategies [242,243]. 

It is crucial to note, that colon and rectal cancer are distinct tumor entities, that require different treatment strategies [25,26] and show relevant disparities in their TIME [27,28]. Therefore, to increase clinical translation, therapy research should account for this variance by using specific tumor models. During our literature research, we observed that some studies on rectal cancer used in fact colon cancer models, which might contribute to low research specificity. In the clinical environment, the interchangeability assumption of the terms CRC and colon cancer, as shown by high rates of misclassification of rectal cancer cases in death records, poses a serious problem [244].

Regarding the applied therapy, in the context of rectal cancer, many of the reviewed studies included CRT and not only RT since this is the current clinical practice. Therefore, it is possible that the observed effects could also be attributed to the chemotherapeutic component of the therapeutic regime or to a synergistic effect of both treatments. Nevertheless, considering that RT might exert a stronger local effect than CT [172], and thereby also have a greater impact on the TIME, it can be speculated that RT might be the major inducer of the reported TIME changes. However, exclusive CT effects on the rectal cancer TIME must be further examined in larger cohorts with state-of-the-art methods. Another relevant factor to be observed, when treating rectal cancer tumors with neoadjuvant radiotherapy, is the interval between irradiation and surgical resection, since multiple immune cells’ counts show a decrease during application of radiation, with a following increase [27,172]. Thus, the TIME can differ according to the time point of resection, potentially influencing the local and abscopal effect.

Based on the literature, combination treatments represent a promising approach to support anti-tumor immunity and overcome mechanisms of resistance by making use of and enhancing the numerous immune-stimulating effects induced by RT, whilst simultaneously blocking immunosuppressive elements. Moreover, research models mimicking the TIME, such as organoid co-cultures containing immune cells [245,246] or the humanization of tumor-bearing animals [247] have gained attention in recent years and are a promising approach to improve rectal cancer research.

## 4. Conclusions

The TIME is a crucial element of the tumor milieu and an important factor to radiotherapy response in rectal cancer, certainly representing a relevant aspect for the successful treatment of this disease. Via activation and suppression of autocrine and paracrine signaling through the release of cytokines and cell–cell interactions, the immune cells are able to exert a plethora of effects on stroma and cancer cells, influencing their survival and ultimately patients’ outcome. Currently, our understanding on the cellular crosstalk between TIME and cancer cells upon CRT, and their role in therapy-induced resistance, is limited. Nevertheless, the available literature suggests that strategies applying combination therapies, particularly involving immunotherapy, are promising approaches [40,41,248].

Therapy research will have a major role in improving personalized medicine, via the establishment of specific experimental tumor models, the identification and classification of well-defined tumor phenotypes and the use of state-of-art methodology. Only in such a manner will we be able to fully comprehend the TIME’s predictive value in rectal cancer.

## Abbreviations

APC	Antigen-presenting cell
CAR-NK	Chimeric antigen receptor-transduced NK cells
CCL	Chemokine C-C motif chemokine ligand
CD	Cluster of differentiation
(n)CT	(Neoadjuvant) chemotherapy
CTL	Cytotoxic T lymphocyte
CRC	Colorectal cancer
(n)CRT	(Neoadjuvant) chemoradiotherapy
CXCL	C-X-C motif chemokine ligand
DAMP	Damage-associated molecular pattern
DC	Dendritic cells
EC	Endothelial cell
EGFR	Epidermal growth factor receptor
FOXP3	Forkhead box P3
HLA	Human leukocyte antigen
ICD	Immunogenic cell death
IFN	Interferon
IHC	Immunohistochemistry
IL	Interleukin
iNOS	Nitric oxide synthase
LAG-3	Lymphocyte-activating gene 3
LAP	Latency-associated peptide
LARC	Locally advanced rectal cancer
LCRT	Long-course radiotherapy
MDSC	Myeloid-derived suppressor cell
MHC	Major histocompatibility complex
MLR	Mixed leukocyte reaction
M-MDSC	Monocytic myeloid-derived suppressor cell
NK cell	Natural killer cell
NLR	Neutrophil to leukocyte ratio
OS	Overall survival
pCR	Pathological complete response
PD-1	Programmed cell death receptor 1
pDC	Plasmacytoid dendritic cell
PD-L1	Programmed cell death ligand 1
PMN-MDSC	Polymorphonuclear monocytic myeloid-derived suppressor cell
(n)RT	(Neoadjuvant) radiotherapy
SCRT	Short-course radiotherapy
SDF-1α	Stromal cell-derived factor-1 alpha
slanMos	6-sulfo LacNAc-expressing monocytes
SOX2	SRY-box transcription factor 2
STAT3	Signal transducer and activator of transcription 3
TAM	Tumor-associated macrophage
TGF-ß	Transforming growth factor-ß
Th cell	T helper cell
TIL	Tumor-infiltrating lymphocyte
TIME	Tumor immune microenvironment
TME	Tumor microenvironment
TNF-α	Tumor necrosis factor α
TNT	Total neoadjuvant therapy
TP	Thymidine phosphorylase
Treg	Regulatory T cell
